# The Fourteenth Annual Meeting of the Southern Dental Association

**Published:** 1882-08

**Authors:** 


### AMERICAN JOURNAL

OF

### DENTAL SCIENCE.

**Vol. XVI. THIRD SERIES—AUGUST, 1882. No. 4**

### ARTICLE I.

### *The Fourteenth Annual Meeting of the Southern Dental Association.*

The annual meeting of the Southern Dental Association was held in the Baltimore College of Dental Surgery, commencing Tuesday morning, August 8th, 1882, at 10 o'clock. A great many of the prominent dental practitioners of the South with quite a representation from the North and West, were present, and the meeting proved to be one of the most interesting ever held by this Association. The Association was called to order by the president, Dr. E. S. Chisholm, of Alabama. An address of welcome was made by Dr. B. Holly Smith, Jr., of Baltimore, and responded to by Dr. J. R. Walker, of New Orleans.

ANNUAL ADDRESS.

Dr. Chisholm delivered the annual address. He said the organization has a mission to perform, or its prime incentive fails, and the result is a useless expenditure of time and means. Its object is to elevate dentistry, improve the hands and hearts of those who practice it, and tender the boon of greater usefulness to humanity. This body, systematically provided with resources for investigation, seeks the development of hidden truth. A dental society should not be a piece of machinery glued together by virtue of its age, number of its members, the name and character it gives, nor the honors it confers. Neither should it be the salve of conventionality nor the servant of autocracy. A dental society should be the recognized vehicle of a progressive age, and fully awake to the spirit of modern advancement. It does not matter how perfect a dental organization we may have unless it may be applied to its legitimate use; unless it fulfils the mission intended it becomes a weight and discouragement, and ultimate abandonment of effort is inevitable. The establishment of a better social feeling among the brotherhood is of no minor import in the work before them. What more befitting spot on God's green earth, he asked, could dentists come to than the Monumental City—Baltimore—the Athens of dental lore, whence germinated our honored profession? This, too, the realized, identical old Baltimore College of Dental Surgery, whence disseminated that spontaneous germ that has within the short period of a few years grown to the magnitude of a dignified and liberal profession. The time-honored college, the founders of which were the first to launch forth upon a sea of uncertainty in search of a new profession, now stands a glorious monument of a demonstrated fact, establishing the verity of and the necessity of its existence. We can claim to have attained that elevation that makes us public benefactors. A growing interest in behalf of the Association is being awakened throughout the southern, central and even some of the northern States. Its membership is increasing by the induction of much of the bone and sinew of our ranks. The peculiar feature of this session will be the extensive and elaborate clinic instruction, conducted under the ablest clinical corps ever present at any of its meetings.

The following members answered to their names at roll call immediately after organizing, very many coming in afterwards and during the subsequent sessions:

Dr. E. S. Chisholm, Tuscaloosa, Ala., President: Dr. W. H. Hoffman, Charlotte, N. C., Recording secretary; Drs.

L. D. Carpenter, Atlanta ; R. B. Winder, Baltimore ; R. Finly Hunt, Washington ; J. R. Walker, New Orleans ; F. J. S. Gorgas, J. H. Harris, and Geo. W. Massamore, of Baltimore ; H. J. McKellops, St. Louis ; J. B. Hodgkin, Washington ; V. E. Turner, North Carolina ; J. C. Uhler, Baltimore ; J. B. Patrick, South Carolina ; Geo. B. Steel, Virginia ; M. W. Foster, Baltimore ; T. M. Allen, Alabama ; J. P. Holmes and J. H. Coyle, Georgia ; W. A. Mills, Baltimore ; W. R. Bull, South Carolina ; H. A. Lowrance, treasurer, and W. W. Ford, of Georgia ; J. A. Hurdle, of North Carolina ; Geo. F. Keesee and J. F. Thompson, of Virginia; W. H. Atkinson, of New York.

The following names were presented by the Executive Committee and elected as members of the Association :

Drs. L. T. Caughey, Maryland ; B. Holly Smith, R. B. Winder, Jr., Wm. B. Finney, Benjamin Flannigain, Baltimore ; C. L. Alexander, N. C.; John Miller, New York ; William Townes Hicksford, Virgania ; A. F. King, New York ; A. D Barrett, Norfolk, Va. ; D. E. Everett, Raleigh, N. C. ; W. W. Freeman, Maryland, B. M. R. Hopkinson, Baltimore, ; E. R. Rust, and D. Rust, Alexandria, Va. ; J. M. Riggs, Hartford, Conn. ; E. D. Hamner, Galveston, Texas ; G. B. White, Chester, S. C. ; Corydon Palmer and Delos Palmer, New York ; Henry S. Abendschein, Baltimore ; F. W. Slueds, Hampton, Va. ; T. H. Parramore, Baltimore ; R. A. Holliday, Georgia.

Dr. E. Parmly Brown was received as a delegate from the Dental Society of the State of New York. Visiting members who were extended a cordial welcome : Dr. Chas. R. Butler, Cleveland, Ohio ; Dr. E. Parmly Brown, Flushing, N. Y. ; W. G. A. Bonwill, Philadelphia ; Dr. Haskell, Chicago ; Dr. H. H. Dwinelle, of New York.

Drs. Carpenter, Walker and Hunt, were appointed a committee on membership.

On motion of Dr. Allen, the medical profession of Baltimore were invited to attend the meetings. The committee appointed to revise the constitution of the Association, Dr.

F. J. S. Gorgas, chairman, submitted a report, which was considered by sections, and elicited much discussion, occupying the time of the Association to the hour of adjournment.

Adjourned until Wednesday morning at 9 o'clock. No night session being held.

WEDNESDAY AUGUST 9TH—SECOND DAY.

The Association reassembled at 9 A. M., Dr. E. S. Chisholm, President, Dr. W. H. Hoffman, Secretary.

This day was devoted to clinics in operative and mechanical dentistry and to the delivery of clinical lectures. Those who lectured were among the most prominent practitioners of dentistry in the country, some of them men who have written valuable works on the subject. The object in delivering the lectures was to better demonstrate what has been written, as it was stated a much clearer idea could be given in that way than in written essays. The clinics were given for the purpose of demonstrating the mode of operating by men of much practical experience, and much valuable information was imparted. Dr. L. P. Haskell, of Chicago, during a clinic in mechanical dentistry, made a full continuous gum set of teeth, and his skillful workmanship was closely watched by other experts. Clinical lectures were given by Dr. J. M. Riggs, of Hartford, Conn., on "Riggs Disease;" by Dr. W. G. A. Bonwill, of Philadelphia, on "Bonwill s Crowns," &c.; by Dr. H. J. McKellups, of St. Louis, and Dr. W. H. Dwinelle, of New York, on "Irregularities." The two latter attended the Medical Congress in London a year ago, and in their lectures gave the result of their investigations while attending that body. Clinics in Operative Dentistry were given by Dr. Corydon Palmer, of New York; Dr. C. R. Butler, of Cleveland, Ohio, and Dr. E. Parmly Brown, of Flushing, New York. Prof. James H. Harris exhibited some gold fillings in the mouth of Dr. James H. Tucker, of Va. which were inserted nearly seven years previously, and which were pronounced by all who saw them to be some of the very best operations ever performed.

EVENING SESSION.

A business meeting was held and the consideration of the constitution was again taken up. It was decided to divide the territory of the Association into four districts, as follows: No. 1—Maryland, Delaware, District of Columbia, Virginia, West Virginia and North Carolina. No. 2—South Carolina, Georgia, Alabama, Mississippi and Florida. No. 3—Louisiana, Texas, Arkansas, New Mexico, Arizona and Indian Territory. No. 4—Kentucky, Tennessee and Missouri. The president, second vice-president and executive committee is to be selected from the district in which the next meeting is to be held; the remaining four vice-presidents to be selected from the three remaining districts in order, and one—the first—from the country at large. The constitution was finally adopted as a whole.

Dr. Wm. H. Dwinell, of New York city, was elected an active member of the Association.

Dr. Hunt, from the committee on the revision of the roll of membership, submitted a report which was adopted. A large number, the report said, had not responded to a circular sent out as to whether they intended to remain as members of the association.

Dr. J. F. Coyle, of Georgia, made an address in behalf of the *Southern Dental Journal.* Dr. Coyle spoke of the importance of printing the thoughts adduced at assemblages of this kind, so as to be understood throughout the profession.

Dr. F. J. S. Gorgas, Dean of the Dental Faculty of the University of Maryland, made an instructive address on the subject of Dental Education. Dr. Gorgas said that an educated brain and hand contributes to the wealth of a country can be as applicably applied to dentistry as any other of the professions. Education is a labor-saving process. There was a certain amount of incompetency among dental practitioners, as in all other professions, which escapes through our colleges. That those who were engaged in dental teaching could not conceal from themselves that it would be desirable to raise the average level of dental acquirement, skill and capacity, and the question is what measures will best promote a better professional education. And while we should inculcate a high standard of knowledge, and bid God speed to progress in an age of science like the present, there is danger that the dental student will be drawn from what is practical, useful, and even essential by well meant enthusiasm. We should endeavor to direct or limit progress by considerations of its relative utility. We must guard against all that mistakes novelty for value, or flatters by a suggestion of exclusiveness in its pursuit. Nearly all dental colleges as well as medical have a board of trustees, but their affairs are administered by the professors, on whose efforts their success wholly depends. Neither a University nor a Board of Trustees or Regents can safely ignore the advice of the teachers and assume more than a general supervision. The object of all dental schools should be to supply practitioners thoroughly competent in all common dental matters, able first to identify and then to treat therapeutically and mechanically the local diseases and lesions of the dental structures, and be free from the prejudices and errors bequeathed by an earlier age of our art. If Dental Colleges could be governed on the principle that it is better to turn out a few graduates educated to a certain standard than a large number not educated quite so well, then a radical change in the present plan of dental teaching would be demanded. But at the same time great care would have to be exercised lest such a system should prove exclusive or impracticable to the many. Good judgment is more to be desired in a dental student than great learning. Any professor will say that it is better to instruct *de novo* than to eradicate opinions inculcated by incompetent preceptors. A general knowledge of anatomy is imparatively demanded in the dental student, and a knowledge of physiology is also necessary. Modern chemistry is the basis of materia medica. A knowledge of general surgery is of great advantage to dental surgeons. Dr. Gorgas said that the establishment of dental departments in universities of medicine was an important movement—a department not of lectures on dentistry only, but where the student has the advantage of clinical instruction and operative dentistry and oral surgery.

The mere establishment of one or more dental chairs in a University for the purpose of securing the delivery of lectures on dental subjects, and which is not supplemented by the necessary clinical instruction, would fall far short of the requirement, mechanical talent being one of the first requisites to success in the practice of dentistry. But the case is materially different where a Dental Department as perfect as possible in all its appointments, having its separate dental faculty, its Infirmary and Laboratory and Lecture Hall, independent in its government, is established in connection with a reputable Medical University, wherein the dental student has all the advantages of the medical student in addition to those of his own particular specialty. Dental students may then gain a broader medical education, the benefit of which in the practice of dentistry is beyond denial.

Dr. J. H. Coyle, of Georgia, read a very interesting paper on the same subject of Dental Education. He referred to the fact that dentistry could not be taught by others than by dentists. [Applause.] He was not opposed to dental departments in medical colleges where they were *bona fide*, and thought in the future they would largely control dental education.

Dr. J. B. Hodgkin, of the Baltimcre College of Dental Surgery, on the same subject, argued that it was not necessary for a man to know all about the intricacies of the medical profession or to know all about the human system to become a good dentist. There was, he said, too many departments now about a medical college, and he did not believe in putting another suckling there without a nipple.

Adjourned until Thursday morning.

THURSDAY MORNING AUGUST 10TH—SECOND DAY.

The Association met at 9 A. M. prior to which time clinics were held in celluloid by Dr. Evans, of Washington, D C., and in continuous gum work by Dr. L. P, Haskell, of Chicago. Dr. Charles L. Steel and Dr. L. M. Cowardin of Richmond, Va. were elected active members. Drs. H. J. McKellops, J. M. Riggs and J. H. Coyle were appointed a committee on dental appliances. Dr. R. Finly Hunt, of Washington, made an address explanatory of electro-plating, and Dr. W. H. Atkinson spoke of an improved base for artificial teeth. Dr. Atkinson said that to substitute any part of the human body was an art of the highest order.

Officers for the ensuing year were elected as follows: President, Dr. L. D. Carpenter, of Atlanta, Ga.; First Vice President, Dr. J. M. Riggs, of Harford, Conn. : Second Vice President, Dr. R. A. Holliday, Atlanta, Ga. ; Third Vice President. Dr. J. R. Walker, New Orleans, La. ; Fourth Vice President, Dr. H. J. McKellops, St. Louis; Fifth Vice President, Dr. J. F. Thompson, Fredericksburg, Va.; Corresponding Secretary, Dr. J. P. Holmes, Macon, Ga. ; Recording Secretary, Dr. W. H. Hoffman, Charelotte, N. C. ; treasurer Dr. H. A. Lowrance, Athens, Ga. ; Executive Committee, Drs. J. H. Coyle, T. M. Allen, J. B. Patrick, R. A. Holliday and A. G. Bouton.

Dr. George F. Keesee, President of the Virginia State Dental Association, was received as a delegate from that Association. Atlanta, Ga., was selected as the next place of meeting. A paper on "Dental Education," prepared by Dr. W. P. Dunlap, of Alabama, was read by Dr. T. M. Allen. The paper stated that some thirty years ago there was but one dental college in existence—the Baltimore Dental College. Reference was made to the rapid increase of dental colleges, and in some instances diplomas were granted when not a chair in the college issuing them is filled nor a student within their walls.

Dr, Wm. A. Mills, of Baitimore, referring to the paper read on Dental Education, said that he differed with Dr. Hodgkin, who claimed that all that was necessary to make a successful dentist was an experienced eye and a skilled hand. If he believed that, he would resign his connection with dental associations, go home and put out a sign "Cheap teeth made by steam." &c. More was wanted than to be a mere machine. A medical education was one of the essential things necessary in order to be a competent dentist.

Dr. J B. Patrick, of Charleston, S. C., read a very interesting paper on "Fætal Medication—the true Hygiene Prophylaxis of Dentition." Dentists should endeavor, he said, to impress upon women that the health and well-being of children depended much upon them. Children are fed with food they cannot assimilate nor digest. Dr. Patrick argued that the health and habits of the maternal parent had much to do with the proper condition of the teeth of their offspring.

Dr. Atkinson, of New York, followed in an interesting scientific discussion of the matter contained in the paper, and became so warmed up on the subject that he took his coat off and spoke in his shirt sleeves. Dr. Atkinson spoke of the importance of giving the mother such food as she requires, and as much of it as she needs, and if she wants pickles, give her pickles, [laughter;] and if children want to chew on gravel or roots, let them do whatever their natures require. [Applause.]

Dr. E. Parmly Brown, of New York, read a paper on "Operative Dentistry." He said the colleges number about a score, the societies many scores, and the journals almost another score, and it depends upon the student himself whether he gets the knowledge demanded or not, and when he gets that knowledge to do his work thoroughly. [Applause.] Thoroughness not only meant a gold filling—a solid gold filling well anchored margined and contoured properly—but it meant all this and more too; to be thorough in all the branches of the profession, and to charge sufficiently to obtain thoroughness. [Applause.] A vote of thanks was tendered Dr. Brown.

Dr. W. W. Ford, of Macon, Ga., spoke of the difficulty of obtaining sufficient pay in his part of the vineyard. Time should be taken to do the work thoroughly, and the pay should be sufficient. The Doctor cited as an instance a lady who complained of his charge for filling a tooth, and said that she would rather lose the tooth than pay so much. He at once offered to give his time and work for nothing and to extract the tooth without pay. The lady preferred paying the bill to losing her tooth. [Laughter.]

Dr. D. McFarlan, of Washington, favored charging well, and then doing the work thoroughly.

Prof. Hodgkin and Dr. Riggs spoke of the use of anæsthetics in the practice of dentistry.

A paper prepared by Dr. H. M. Grant, of Abingdon, Va., was read by Dr. Hunt.

A committee on constitution and by-laws was appointed, consisting of Dr. Winder, of Maryland, Dr. Coyle, of Georgia and Dr. Turner, of North Carolina, to make further revision if deemed necessary and to report next year.

Adjourned to meet in Atlanta, Georgia, on the second Tuesday in August, 1883. The members of the Association were given a complimentary excursion on Friday, by the dentists of Maryland and the District of Columbia to Annapolis and Tolchester, on the steamer Matilda.

During the session, an invitation was extended to the members of the Association to visit the new Dental Infirmary and Laboratory Building and Lecture Halls, Museum, etc., of the University of Maryland, which was availed of by the majority of those in attendance, and who expressed their satisfaction with the complete arrangements provided for the instruction of dental students, The following standing committees were appointed by the President, Dr. L. D. Carpenter, to report at the next meeting to be held in Atlanta in 1883:

*Publication.*—Drs. W. H. Hoffman, of N. C., Chairman; C. C. Patrick, S. C.; R. A. Holliday, Ga.

*Dental Education.*—Drs. J. P. H. Brown, Ga., Chairman; C. L. Steel, Va.; A. C. Ford. Fla.; W. H. Marshall, Ga.

*Dental Hygiene.*—Drs. R. F. Hunt, D. C., Chairman; E. S. Chisholm, Ala.; G. B. White, S C.; R. A. Freeman, Ga.; S. G. Holland, Ga.

*Pathology and Therapeutics.*—Dr. J. M. Riggs, Conn., Chairman,; J. B. Patrick, S. C.; G. F. S. Wright, S. C.; G, J. Frederick, La.; E, L. Hunter, N. C.

*Histology and Microscopy,*—Drs. W. H. Atkinson, N, Y.; Chairman; W. W. H. Thackston, Va.; H J. McKellops, Mo.

*Chemistry.*—Drs. J. R. Walker, of La., Chairman; W. H. Dwinelle, New York; H. J. Royal, Ga.; D. Hopps, Ga.

*Operative Dentistry.*—Drs. J. H. Harris, Md., Chairman; T. Chupein, Pa.; A. G. Bouton, Ga.; W, S. Brown, S. C.; L. B. Barfield, Ga.; T. M. Allen, Ala.

*Mechanical Dlntistry.*—Drs. J. B. Hodgkin, D. C Chairman; W. W. Evans, D. C.; H. H. Keith, Mo.; L. M. Cowardin, Va,; M. S. Dobson, Ga.; J, C. Uhler, Md,

*Dental Literature.*—Drs. F J S. Gorgas, Md., Chairman; A. O Rawls, Ky.; A. F. McLain, Cal.; W. C Wardlaw, Ga.

*Voluntary Essays.*—Drs. N. W. Kingsley, N, Y,,. Chairman; E. D. Hamner, Tex.; D. E. Everitt, N. C.; T. C. Moore, S. C.; J. S. Milbourne, Miss.; J. T. Calvert, S. C.; S. H. Henkle, Va.; J. G. McAuley, Ala.

*Dental Appliances.*—Drs. W. G. A. Bonwill, Pa., Chairman; W. W. Ford, Ga.; M. W. Foster, Md.; M. A. Bland, N. C.; S. A. White, Ga.

